# When to use frovatriptan in migraine?

**DOI:** 10.1007/s10194-011-0341-5

**Published:** 2011-04-22

**Authors:** Peer Carsten Tfelt-Hansen

**Affiliations:** Department of Neurology, Danish Headache Center, Glostrup Hospital, University of Copenhagen, Glostrup, Denmark

Sir,

In two cross-over randomized controlled trials (RCTs) published in the Journal of *Headache and Pain,* frovatriptan 2.5 mg had a similar efficacy to that of rizatriptan 10 mg [1] and almotriptan 12.5 mg [2]. In both RCTs preference, the primary efficacy measure was quite comparable as were pain free and headache relief after 2 h, and sustained pain free for 2–48 h [1, 2]. In contrast, in a systematic review of triptans [3n] the mean therapeutic gain (active minus placebo) for headache relief at 2 h was 19% (95% CI 16–22%) for frovatriptan 2.5 mg, whereas it was 33% (95% CI 31–35%) for sumatriptan 100 mg, 34% (95% CI 30–37%) for zolmitriptan 2.5 mg, 36% (95% CI 32–39%) for rizatriptan 10 mg, and 27% (95% CI 20–33%) for almotriptan 12.5 mg. The superiority of sumatriptan 100 mg versus frovatriptan 2.5 mg was confirmed in a not fully published large (*n* = 1196) RCT in which the headache relief rates were 47 and 37%, respectively [4, 5]. The recurrences rates for frovatriptan (25%) and sumatriptan (31%) were similar.

Migraine patients in these RCTs [3, 4, 6] waited until the headache was moderate or severe whereas patients in the cross-over RCT were instructed to treat the migraine attack as early as possible [1, 2]. Data on headache severity at the time of treatment were not reported in the papers on these two RCTs [1, 2]; a peculiar omission in papers on acute migraine RCTs. In addition, patients not responding to triptans were excluded and the patients in the two cross-over trials were, therefore, most likely less afflicted by migraine than the ones participating in the parallel group RCTs in which such patients were not excluded. The most likely reason for the comparable efficacy of frovatriptan and zolmitriptan [7], rizatriptan [1] and almotriptan [2] in these cross-over RCTs is, however, the very early use of test treatment.

In conclusion, frovatriptan is for moderate and severe headache not the triptan of first choice (therapeutic gain for frovatriptan is only 19 vs. 27–36% for other triptans, see above). However, if the migraine patients are able to treat their attacks very early, and have not demonstrated inadequate response to other triptans, then frovatriptan is most likely a good alternative to other triptans.

Yours sincerely

Peer Carsten Tfelt-Hansen
